# Proteomic analysis of the *Plasmodium* male gamete reveals the key role for glycolysis in flagellar motility

**DOI:** 10.1186/1475-2875-13-315

**Published:** 2014-08-13

**Authors:** Arthur M Talman, Judith H Prieto, Sara Marques, Ceereena Ubaida-Mohien, Mara Lawniczak, Mark N Wass, Tao Xu, Roland Frank, Andrea Ecker, Rebecca S Stanway, Sanjeev Krishna, Michael JE Sternberg, Georges K Christophides, David R Graham, Rhoel R Dinglasan, John R Yates, Robert E Sinden

**Affiliations:** Division of Cell and Molecular Biology, Imperial College, London, UK; Department of Microbial Pathogenesis, Yale University School of Medicine, New Haven, CT 06510 USA; The Scripps Research Institute, La Jolla, CA 92037 USA; Chemistry Department, Western Connecticut State University, Danbury, CT USA; Department of Molecular & Comparative Pathobiology, Johns Hopkins School of Medicine, 733 N Broadway, Baltimore, MD 21205 USA; The Centre for Bioinformatics, Department of Life sciences, Imperial College, London, SW7 2AZ UK; Centre for Molecular Processing, School of Biosciences, University of Kent, Canterbury, Kent, CT2 7NH UK; Dow AgroSciences LLC, 9330 Zionsville Road, Indianapolis, IN 46268 USA; Research Centre for Infectious Diseases, University of Würzburg, Röntgenring 11, 97070 Würzburg, Germany; Bernhard Nocht Institute for Tropical Medicine, Bernhard-Nocht-Str 74, 20359 Hamburg, Germany; Centre for Infection, Division of Cellular and Molecular Medicine, St George’s, University of London, Cranmer Terrace, London, SW17 0RE UK; W Harry Feinstone Department of Molecular Microbiology & Immunology, The Johns Hopkins Malaria Research Institute, 615 N Wolfe Street, Baltimore, MD 21205 USA

**Keywords:** Gamete, *Plasmodium*, Glycolysis, Flagellum, Energy metabolism

## Abstract

**Background:**

Gametogenesis and fertilization play crucial roles in malaria transmission. While male gametes are thought to be amongst the simplest eukaryotic cells and are proven targets of transmission blocking immunity, little is known about their molecular organization. For example, the pathway of energy metabolism that power motility, a feature that facilitates gamete encounter and fertilization, is unknown.

**Methods:**

*Plasmodium berghei* microgametes were purified and analysed by whole-cell proteomic analysis for the first time. Data are available via ProteomeXchange with identifier PXD001163.

**Results:**

615 proteins were recovered, they included all male gamete proteins described thus far. Amongst them were the 11 enzymes of the glycolytic pathway. The hexose transporter was localized to the gamete plasma membrane and it was shown that microgamete motility can be suppressed effectively by inhibitors of this transporter and of the glycolytic pathway.

**Conclusions:**

This study describes the first whole-cell proteomic analysis of the malaria male gamete. It identifies glycolysis as the likely exclusive source of energy for flagellar beat, and provides new insights in original features of *Plasmodium* flagellar organization.

**Electronic supplementary material:**

The online version of this article (doi:10.1186/1475-2875-13-315) contains supplementary material, which is available to authorized users.

## Background

Development of the haploid malarial parasite in the vertebrate host culminates with formation, in the blood of developmentally arrested sexual forms, the gametocytes. Transmission to the invertebrate vector requires these cells to undergo gametogenesis and fertilization within the blood meal of the engorged female mosquito.

Male gametes of *Plasmodium* are one of the simplest of all eukaryotic cells. They are composed of very few recognizable cellular entities, among which are a nucleus containing a condensed haploid genome, an axoneme attached to a modified basal body, and a plasma membrane [1].

The very short life span of the male gamete (30–40 min) [2] can be divided into different phases. The first is exflagellation, which consists in the budding of eight male gametes from each parental cell. Exflagellation requires vigorous flagellar beating, both to complete cytokinesis and to liberate the gametes. Exflagellation is followed by free swimming, this consists of two types of flagellar beat: fast (5 beats/sec) and slow (1 beat/sec) [2]. When free-swimming male gametes encounter a female, they adhere, this is accompanied by ‘rubbing’ of the male gamete on the female surface [2]. The last phase is fusion of the male and female gametes, a process characterized by a period of intense flagellar beating that continues even after the male axoneme and nucleus enters the female cytoplasm [2].

The *Plasmodium* axoneme possesses a conserved 9 + 2 microtubule doublet organization, with outer and inner dynein arms and a central apparatus. However, it has been shown to assemble in a unique fashion as compared to other known eukaryotic flagella. The assembly occurs in the cytoplasm of the activated parental male gametocytes and does not rely on intraflagellar transport (IFT) [3, 4]. Flagellar activity requires significant ATP production to drive the dynein motor [5] and consequent sliding of the adjacent microtubule doublet [6]. *Plasmodium* male gametes do not possess mitochondria [7–9] and it is unknown how they produce sufficient ATP to drive their intense flagellar beat.

This study reports the first proteomic analysis of malaria male gametes, compares it with the previously published male gametocyte proteome [10], and identifies novel aspect of flagellar assembly and composition and establishes glycolysis as the exclusive source of energy for microgamete motility.

## Methods

### Male gamete purification

A method for the purification of microgametes of *Plasmodium gallinaceum* was previously published [11], it was adapted to the rodent parasite *Plasmodium berghei. Plasmodium berghei,* strain ANKA clone 2.34, was maintained by cyclic passage in six to eight week-old female Tuck’s Original (TO) mice (Harlan, UK). Mice were injected intraperitoneally (ip) with 0.2 ml of 6 mg/ml phenylhydrazine (BDH Chemicals Ltd, UK) (to induce hyper-reticulocytosis) two to three days prior to infection.

At day 4 post-infection, mice were injected ip with 150 μl sulphadiazine (4 mg/ml) to remove asexual parasites [12]. After 24 hours, parasites were harvested and gametocytes were allowed to activate in exflagellation medium (RPMI 1640 (Sigma, UK) supplemented with 25 mM Hepes (Sigma, UK) and 100 μM xanthurenic acid (Sigma, UK), pH 7.4) for 20 min. The parasites were pelleted at 500 g for 5 min and resuspended in their own supernatant; the parasites were pelleted again and the supernatant (containing extracellular male and female gametes) was harvested and spun at 10,000 g for 7 min. The pelleted samples were incubated at room temperature for 10 min, the supernatant (now containing male gametes that had swum from the pellet) was harvested and spun at 12,000 g for 10 min at 4°C and washed twice in PBS. Samples were immediately stored at -80°C. The purity and quantity of the sample was determined by microscopy following Giemsa (Sigma, UK) staining.

### Proteomics

As described previously [13, 14], pellets were treated with a 5× solution of Invitrosol (Invitrogen, UK) and heated to 60°C for 5 min, vortexed for 2 min and sonicated for 1 hr. The solution was diluted to 1× Invitrosol with 100 mM Tris buffer (pH 8.5). Samples were reduced and carboxyamidomethylated, followed by digestion with endoproteinase Lys-C for 6 hr. The solution was diluted to 4 M urea with 100 mM Tris buffer and further digested with trypsin. Peptide mixtures were analysed by MudPIT as described previously [15, 16] with modifications. An Eksigent HPLC coupled directly to a Finnigan LTQ-Orbitrap mass spectrometer (ThermoFisher, San Jose, CA, USA) equipped with a nano-LC electrospray ionization source [17] was used and peptide mixtures were resolved by strong cation exchange liquid chromatography upstream of reverse-phase liquid chromatography as described [18].

Peptides eluted from the microcapillary column were electrosprayed directly into an LTQ-Orbitrap mass spectrometer (Thermo Fisher, USA) with the application of a distal 2.4 kV spray voltage with an inlet capillary temperature of 250°C. A cycle of one full-scan mass spectrum (300–2,000 m/z) followed by three data-dependent (window of 2 m/z) CID MS/MS spectra at a 35% normalized collision energy was repeated continuously throughout each step of the multidimensional separation.

Fully automated 11-step chromatography was carried out on each sample. From the resulting data, poor quality spectra were removed using an automated spectral quality assessment algorithm [19]. Raw files were converted to mxzml format using MSConvert (ProteoWizard). The 339,763 MS/MS spectra from three biological replicates (GametesRep1: 102,987 MS/MS spectra, GametesRep2: 110,826 MS/MS spectra, and GametesRep3: 125,950 MS/MS spectra) were searched by PepArML [20] meta search engine. Mascot 2.2 [21], OMSSA 2.1.1 [22], X!Tandem 2010.01.01.4 [23] with K-score 2010.01.01 [24], and S-score 2010.01.01.4 plugins, Inspect 20110313 [25] and MyriMatch 1.5.8 [26] search engines were used within PepArML with the following parameters: semi-specific trypsin digestion, one missed cleavage, precursor tolerance 10 ppm and fragment tolerance 0.5 Da and maximum 4 charge state, fixed carbamidomethyl modification of cysteine and variable oxidation of methionine. A custom-built FASTA database (51,833 sequences) with *P. berghei* (GeneDB, 2013), Human (SwissProt, 2013) and Mouse (SwissProt, 2013) sequences were used. Search results from all search engines were combined and a combination false discovery rate (FDR) was reported. Combination was performed using an unsupervised machine-learning strategy which estimates peptide identification FDR from reversed decoy searches [27]. The search results were analysed for result interpretation in MASPECTRAS 2.0 [28] with 1% spectra FDR and 1% peptide FDR, a minimum of two peptides for a protein, and at least five amino acids for a peptide. The identified proteins from three different biological runs were pooled for result interpretation. Protein identifications passing thresholds from human and mouse were excluded and the remaining *P. berghei* protein identifications were clustered according to shared peptides; clustering was performed because many proteins were isoforms, splice variants or fragments of a representative protein in each protein cluster. The data analysis system meets all standards regarding the minimum information about a proteomics experiment (MIAPE). The mass spectrometry proteomics data have been deposited to the ProteomeXchange Consortium [29] via the PRIDE partner repository [30] with the dataset identifier PXD001163.

### Bioinformatics

Genomic and proteomic data from malaria parasites were obtained either from original papers or from PlasmoDB [31] or the Sanger Institute [32]. Subcellular localization was predicted by a combination of identifying transmembrane proteins and then using a consensus of different subcellular localization predictions. Transmembrane proteins were identified using SCAMPI [33], tmHMM [34] and TOPCONS [35]. Subcellular localization predictors used to make consensus predictors were: Wolf psort [36], sherloc2 [37], ESLPred2 [38], Euk mPLoc [39] and Cello [40].

Gene ontology (GO) functions were also predicted using a combination of BLAST [41], Pfam [42], Interpro [43] and specialized GO predictors including: ConFunc [44], PFP [45] and FFPRed [46]. Protein structures homologous to the gamete proteins were identified by searching the fold library of the protein structure prediction server Phyre [47]. The enzyme classification [48] and GO functions of the homologous structures were also used to infer the function of the gamete proteins.

For the GO enrichment analysis, the BioConductor package topGO [49, 50] was used in R version 2.10.0. This program identifies significant GO terms by taking the hierarchical structure of GOs into account. To identify significantly over-represented GO terms among the different proteomes, the ‘weight01’ algorithm within topGO was used in which genes are classed as either 0 or 1, using a minimum p-value cut-off of 0.1. All reported p-values are adjusted using a false discovery rate procedure [51].

### Generation of PbHT-myc

One-thousand and thirty bp of the *P. berghei* hexose transporter gene (PBANKA_030250) were amplified in a two-step PCR to insert an EcoRV linearization site for subsequent transfection. AdvantageII Taq polymerase (Takara Bioscience) was used for all amplifications. Primers F (GG*GGTACC*TGGTGTATTGCATCAGTTAT, KpnI site in italics) and MR (GAAAACCTGATATCATACATCCT) as well as MF (AGGATGTAT*GATATC*AGGTTTTC, EcoRV site in italics) and R (TT*GGGCCC*AACTCTTGATTTGCTTATATGTT, ApaI site in italics) were used for the first PCR. The products of these PCRs were then used as templates for the second PCR with primers F and R. The resulting fragment was inserted into vector p0007 (courtesy of J D Raine) to produce *pHTmyc*. This plasmid contains the hexose transporter homology region; two c-myc tags followed by the 3′UTR from *P. berghei dhfr* and the *Toxoplasma gondii dhfr/ts* resistance cassette (see Additional file 1).

Parasite transfection and pyrimethamine selection were performed as described previously using the Human T Cell Nucleofector kit (Amaxa) [52]. Integration of the construct was monitored by Southern blot (see Additional file 1).

### Immunofluorescence

Samples were harvested and resuspended in 4% paraformaldehyde (PFA) (Novagen, UK) and allowed to settle on poly-L-lysine- (Sigma, UK) coated slides overnight at 4°C. The slides were washed once with Tris-buffered saline (TBS) for 5 min and then permeabilized with 0.2% Triton X-100 (Sigma, UK) in PBS. The slides were then washed three times in TBS and incubated in blocking solution (10% goat serum (Sigma, UK) and 3% BSA (Sigma, UK) in TBS) for 45 min and subsequently probed overnight at 4°C with primary antibody in 1% BSA (Sigma, UK) in TBS. Primary antibodies were rabbit anti-myc polyclonal antibody (Cell Signaling, USA) and mouse anti-alpha tubulin (Sigma, UK). The slides were washed three times in TBS and incubated for 1 hr with secondary antibody in 1% BSA (Sigma, UK) in TBS. The secondary antibodies were Alexa-488 conjugated anti-rabbit IgG and Alexa568 conjugated anti-mouse IgG (Molecular probes, UK). The slides were washed three times in TBS and mounted in Vectashield with DAPI (Vector Labs). Preparations were labelled with secondary antibodies alone to verify the absence of non-specific labelling. Parasites were visualized on a Leica SP5 confocal microscope and acquired and analysed with the LAS AF Lite software (Leica, UK).

### Male gamete motility assay

Mice were infected as described above; on day 3 post-infection, tail blood was harvested and resuspended in one volume of exflagellation medium. The parasites were incubated for 10 min to allow exflagellation to take place and were then suspended for a further 10 min at room temperature in three volumes of PBS containing varying concentrations of CM3361, 2-deoxy-D-Glucose (Calbiochem, UK) and D-Glucose (Sigma, UK). The parasites were visualized by phase contrast microscopy for a maximum of 10 min on a Leica DMR microscope. Five-second videos were captured for at least 30 male gametes for each of three biological replicates in each treatment regimen using a Zeiss AxioCam HRC and Axiovision software. Videos were taken at a speed of 13 images per sec. The number of waves per second (wave frequency) was established over 5-sec periods, for gametes that displayed ‘uniform’ beat frequency for the period of observation.

## Results

### Proteomic analysis identifies a male gamete proteome

This method for purifying *P. berghei* male gametes gave yields of 10^7^ gametes for each sample with no microscopically detectable female contamination; the preparations did however contain mouse platelets (2.5 male gametes per platelet) and to a lesser extent erythrocyte ghosts (155.6 male gametes per erythrocyte ghost).

Three samples were prepared and subjected to MuDPIT proteomic analysis. Resulting spectra were searched against the *P. berghei* genome resulting in the identification of 615 unique *Plasmodium* proteins. In order to maximize true positive identifications and minimize false positives, the data was searched with several search engines and the results combined by an unsupervised machine learning combiner. ROC curves from each individual search engine were compared with the combined results from the machine learning combiner [20], using the false positive rates calculated from a random database [27] (see Additional file 2). The ROC curves clearly show the increases in sensitivity and specificity gained by the multiple search and combination. Protein clustering was then done on the basis of shared peptides, which removes most redundant protein identifications [28].

The full list of proteins identified after clustering is given in Additional file 3.

The proteome is likely to be of high quality since all proteins that previously have been localized to the male gamete or associated with a male gamete function were recovered (Table 1). Nevertheless several recovered proteins indicated low-level contamination. The presence of female gametocyte specific proteins (e g, three of the LCCL-containing proteins (CCP1, 3, 5) and Pb47) (see Additional file 3) is surprising as no female gametocytes were observed in the preparation. These proteins have been previously shown to be secreted upon female gametocyte activation and may remain associated with the erythrocyte ghost or with microgametes following female gamete egress. Therefore, presence of female proteins is likely due to either carry over from cellular debris or from erythrocyte ghost contamination. This latter hypothesis is strengthened by the rare presence of *Plasmodium* proteins previously associated with the infected host erythrocyte (e. g. Pb-fam, CLAG) (see Additional file 3).

**Table 1:** **Proteins identified in the proteome for which experimental evidence suggests they play a role in male gamete biology**

Accession	Seq count	Name	Function	Reference
PBANKA_061520	24	CDPK4	activation	[53]
PBANKA_121260	2	HAP2	fusion	[54]
PBANKA_093370	4	MAP2	cytokinesis	[55]
PBANKA_030600	11	P230p	?	[56]
PBANKA_135960	13	P48/45	adhesion	[57]
PBANKA_030610	53	Pb230	adhesion	[58]
PBANKA_143220	12	MDV1	egress	[59]
PBANKA_131270	23	GEST	egress	[60]
PBANKA_091740	3	PF16	flagellar	[61]
PBANKA_103010	8	actin II	flagellar	[62]

*PB* = *P. berghei* accession number; *Seq count* = number of sequences identifying the proteins in the *P. berghei* male gamete proteome.

### In-depth bioinformatics analysis of the male gamete proteome

Automated subcellular localization predictions (see Methods) were manually validated to make overall predictions. Using this approach, localizations were assigned to 581 of the 615 gamete proteins. The proteins were predicted to belong to either one or more locations in the cell, with 30% predicted to be cytoplasmic. The proteins that are currently unannotated were predominantly predicted to be either nuclear, membrane associated, flagellar, or extracellular. This approach was complemented by functional predictions (see Methods) using GO terms (see Additional file 4). The main functions predicted are for roles in transport, DNA/RNA binding and flagellar-associated functions.

### Gene ontology analysis

To obtain an overview of the proteins identified and their putative function, a GO enrichment test was performed. Two enrichment tests were run: one of the male gamete proteome against all GO predictions in the genome, and one of the previously published male gametocyte proteome [10] against all GO predictions in the genome. Results are summarized in Additional file 4.

The male gamete and the male gametocyte exhibit significantly different GO term profiles, some of which mirror prior biological observations:

-  Components of DNA metabolism and DNA replication are significantly represented in the gametocyte, but only components of DNA packaging are present in the microgamete;
-  Constituents of protein translation are more significantly represented in the male gametocyte and less so in the male gamete;
-  Pathways involved in maintaining metabolic homeostasis, heat shock and stress-response are significantly represented in both cell types;
-  Energy metabolism in both cell types is characterized by an very significant enrichment of glycolytic and an under-representation of mitochondrial enzymes (whilst the male gametocyte contains an enlarged mitochondrion [9], the male gamete lacks this organelle [7]).
-  Components of the cytoskeleton and in particular microtubule and dynein-related proteins are represented in both cell types.

### Energy metabolism in *Plasmodium*male gametes

**Figure 1:**
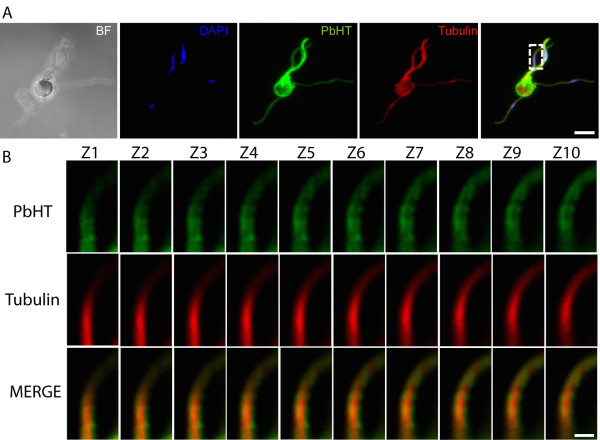
**PbHT localizes to the male gamete surface.** The *P. berghei* HT-myc line was labelled with an α-tubulin antibody (red), an anti-c-myc antibody (green) and stained with DAPI (blue). **(A)** 3-D projection of a male gametocyte in the process of exflagellation. Scale bar 5 μm. **(B)** Enlarged inset from **(A)** displaying the distribution PbHT-myc and α-tubulin in sequential Z stacks (0.1 μm). Scale bar 1 μm. PbHT-myc is abundant and localized to the periphery of male gametes.

To examine energy metabolism in male gametes, the dataset was scanned for metabolic enzymes. Strikingly, all 11 enzymes of the glycolytic pathway and the hexose transporter (PbHT) were present (Table 2). PbHT is the only predicted *Plasmodium* hexose transporter and is expressed ubiquitously in the life cycle [63]. The presence of all molecules participating in glucose fermentation, as well as the result of the GO enrichment test for glycolysis (see Additional file 4) led to the hypothesis that male gametes rely exclusively on glucose metabolism for energy production. To test this hypothesis, a transgenic *P. berghei* line expressing a double c-myc tag on the carboxyl-terminus of the endogenous PbHT was generated [see Additional file 1]. PbHT-myc was labelled and imaged on/in male gametes/gametocytes. Because male gametes are very narrow (0.28 μm), it is challenging to distinguish between cytoplasmic and surface labelling. Confocal microscopy was thus used to enhance the resolution of the signal on the exflagellating microgametocyte (Figure 1A). One can distinguish the surface-association of PbHT in Figure 1B, which displays sequential Z-stacks of PbHT labelling. This observation strongly but not unambiguously suggests that PbHT is a component of the male gamete plasma membrane.

**Table 2:** **Glycolytic enzymes identified in the proteome**

***P. berghei***accession no	Seq count	Spectra	Description
**Glycolytic enzymes**			
PBANKA_121090	6	8	Phosphoglucomutase
PBANKA_121430	38	457	Enolase
PBANKA_092810	23	85	Phosphoglycerate mutase
PBANKA_111770	3	7	Malate dehydrogenase
PBANKA_134010	24	239	LDH L-lactate dehydrogenase
PBANKA_134040	26	144	Oxidoreductase
PBANKA_100880	21	55	Glucose-6-phosphate isomerase
PBANKA_130380	20	102	Triose-phosphate isomerase
PBANKA_130860	47	597	ALDO2 fructose-bisphosphate aldolase 2
PBANKA_132640	53	548	Glyceraldehyde-3-phosphate dehydrogenase
PBANKA_040480	12	27	FAD-dependent glycerol-3-phosphate dehydrogenase
PBANKA_071550	5	11	Phosphoglycerate mutase
PBANKA_112290	17	75	Hexokinase
PBANKA_112560	22	161	Pyruvate kinase
PBANKA_081640	27	65	6-phosphofructokinase
PBANKA_082340	26	122	Phosphoglycerate kinase
**Transporter**			
PBANKA_030250	8	30	HT hexose transporter
**Reference proteins**			
PBANKA_121260	2	2	HAP2 generative cell specific 1
PBANKA_120690	51	656	Beta tubulin

*Seq count* = number of sequences identifying the proteins in the *P. berghei* male gamete proteome; Spectra = number of spectra identifying a protein.

**Figure 2:**
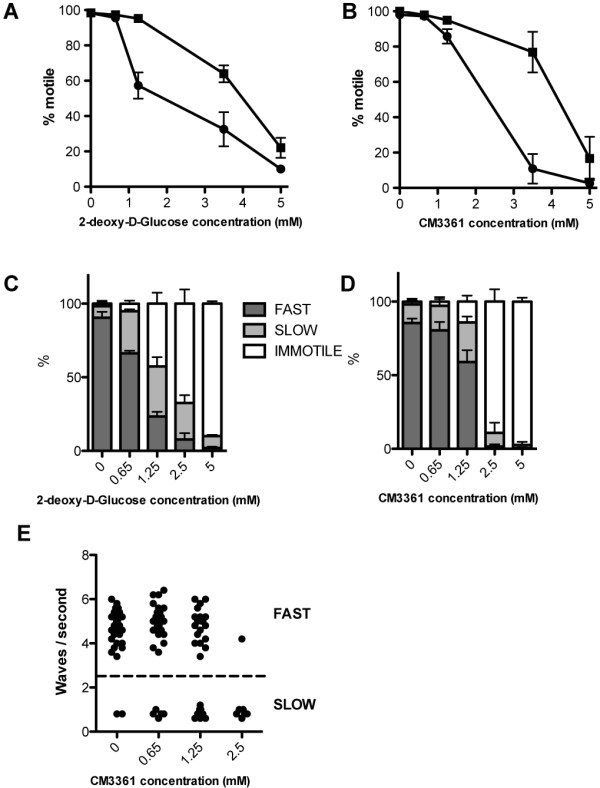
**Male motility is suppressed by glycolytic inhibitors.** The proportion of male gametes exhibiting each class of flagellar beat was determined in the presence of the glucose analogues 2-deoxy-D-glucose (not processed after phosphorylation by hexokinase) **(A)** and CM3361 (competitive inhibitor of glucose transport) **(B)** in the presence  or absence  of excess glucose (10 mM). **(C, D)** Patterns of motility were further categorized into fast beat, slow beat and immotile. **(E)** Values of flagella wave frequencies were measured with different concentrations of CM3361. Frequencies of waves were not altered by the presence of the inhibitor.

To test directly the role of glycolytic fermentation in male gamete motility, two different competitive glycolytic inhibitors were tested: CM3361, a specific inhibitor of PbHT [64], and 2-deoxy-D-glucose (2DG), which is phosphorylated by hexokinase but cannot then undergo glycolysis. Both competitive inhibitors were previously shown to inhibit *in vitro* asexual growth of *Plasmodium falciparum* [64, 65]. As hypothesized, both compounds inhibited male gamete motility in a dose-dependent manner (Figure 2A and B). In spite of the high concentration that was required to achieve inhibition, it was partially rescued by adding excess glucose (+10 mM) (Figure 2A and B), indicating that the inhibition is in all probability specific to glucose metabolism.

Microgametes reportedly exhibit three motility states: fast (~5 waves per sec; Additional file 5; 2.5-5 sec), slow (~1 wave per sec; Additional file 5; 0–2.5 sec) and immotile. The fast beat is essential to both forward movement and fertilization. The work required to accomplish fast beat is reportedly 100 times greater than that required for slow beat [2]. To refine this motility analysis, the number of gametes that exhibit each motility state was scored. Interestingly, beat frequency was not changed by CM3361 (Figure 2E). Instead the proportion of male gametes displaying each motility class (fast beat, slow beat) was considerably reduced. At the lowest 2DG concentrations (0.65 and 1.25 mM), the proportion of motile gametes exhibiting slow beat, as opposed to fast, tripled when compared to controls (Figure 2C). A similar trend is observed for CM3361 (Figure 2D). At higher concentrations of both compounds, most gametes are immotile (Figure 2C and D).

The presence and reproducible detection of all enzymes of the glycolytic pathway as well as the hexose transporter (PbHT) suggest that glucose metabolism is a vital cellular activity in male gametes. The localization of PbHT to the surface of male gametes combined with the observation that glucose transport and metabolism inhibition significantly diminish male motility strongly supports this hypothesis, and are entirely consistent the detection of few mitochondrial proteins. Recognizing prior electron microscopic [1, 7], genetic [66] and protein tagging [9] studies that put beyond doubt the conclusion that the male gamete of *Plasmodium* lacks a mitochondrion, those proteins detected at low abundance must be present simply due to the passive inclusion of cytoplasmic mitochondrial proteins from the parental cell (which has a mitochondrion).

Glycolytic enzymes have been associated with production of ATP for the flagellar dynein motor [67–70] in organisms as diverse as the green alga *Chlamydomonas reinhardtii*, the kinetoplastid *Trypanosma brucei* procyclic form, and mammalian and sea urchin sperm. Different energy management strategies are used by these organisms. For instance, sea urchins rely on a phosphocreatine shuttle to vector an energy flux along the flagella that can rapidly reach the distal end of this long structure [71]. In *Chlamydomonas,* certain glycolytic enzymes have been found in association with the axoneme or with the membrane surrounding it, indicating that at least some steps of glucose fermentation are taking place in very close proximity to the flagella [68]. PbHT was abundant and evenly spread on the whole male gamete, that glycolysis could take place in the little cytoplasm surrounding the axoneme or the larger volume within the axoneme itself. The net balance of fermentation of one glucose molecule is two ATP, two pyruvate, two NADH and two H + molecules. The presence of lactate dehydrogenase implies that NADH is oxidized back to NAD + for continued use by GAPDH using two pyruvate and two protons. The product of this reaction is lactate, which is an acidifying factor in the cytoplasm. The resultant impact on cytoplasmic pH will have to be addressed by the cell, however surprisingly no genomic or, here, proteomic evidence for the presence of a conventional lactate exporter was found.

There was no correlation between the availability of energy (i e, concentration of inhibitor, or the presence of ‘added’ glucose) and flagellar beat frequency, but rather male gametes were seen to change the proportions of time spent in each activity (fast beat, slow beat and immotile) with changing glycolytic activity. While the energy-intensive fast beat results in efficient translocation, the biological significance of the slow beat has not been established. The observation that more slow beating male gametes are seen as inhibitor concentrations rise suggests a link between slow beat and decreased energy availability. One could speculate that this behaviour is a low-consumption holding pattern allowing the balance of metabolites (i e, import of glucose, export of glycolytic by-products) to be restored in order to allow further fast beating. Sinden and Croll [2] observed that the slow beat behaviour was more prevalent as the gametes aged, even in conditions where glucose concentrations would not have changed significantly (*in vitro* activation in high glucose medium). Male gametes may thus become less able to maintain homeostatic balance as time passes because of senescence. It is perhaps unsurprising to note that male gametes have a finite lifespan that correlates with overall beating time; they may be kept (immotile) on ice and preserve beating activity for several hours, but following >40 min of active beating, male gametes invariably become immotile.

### Flagellar organization

**Figure 3:**
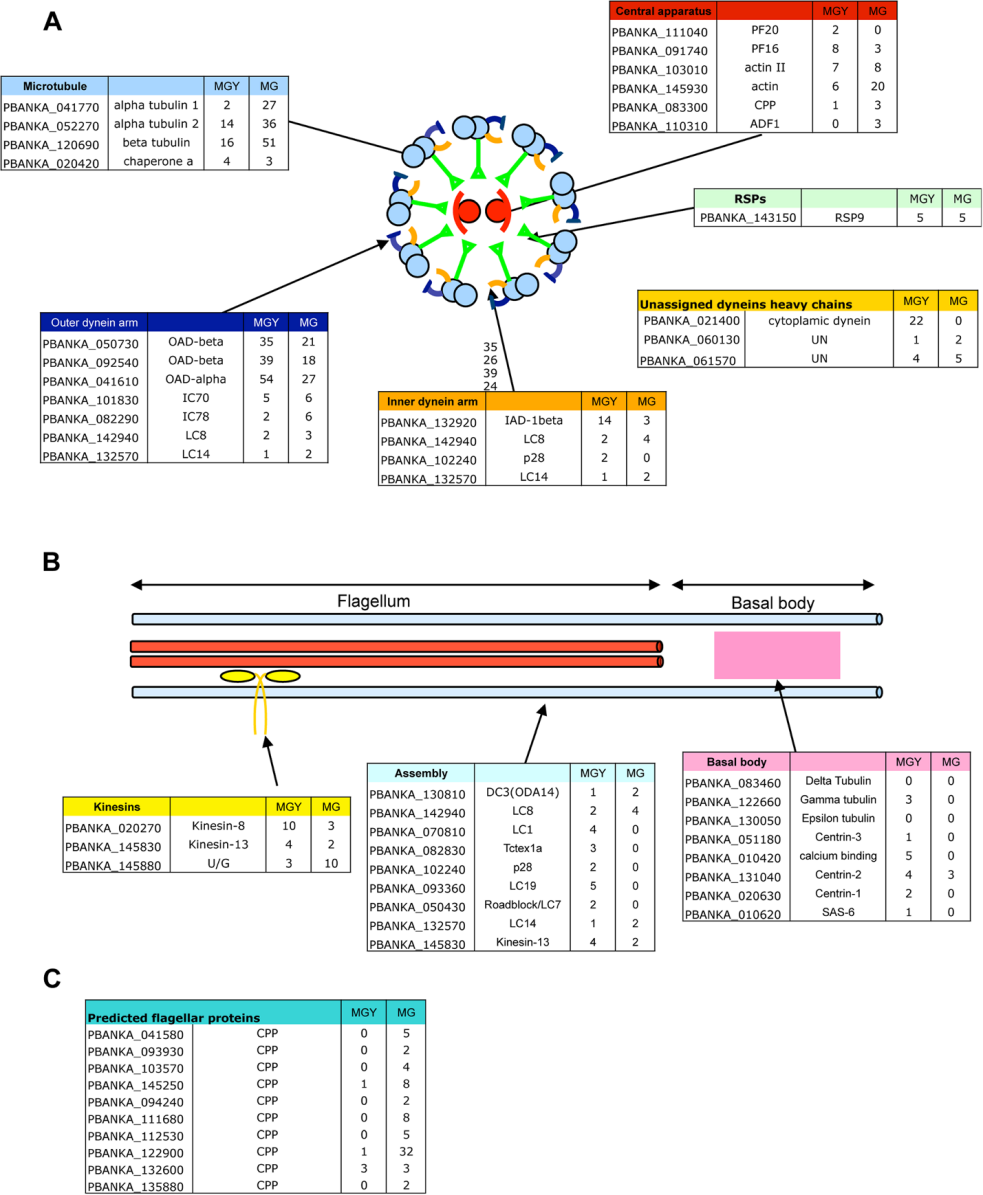
**A model of the** ***Plasmodium*** **flagellum and proteins comprising it. (A)** A cross section of a typical axoneme is shown (as observed in *Plasmodium* and other eukaryotic species). Proteins that were present in the male gametocyte (MGY) or male gamete (MG) proteomes are shown and their sequence count is given. It is noteworthy that the sequence counts originate from different datasets from distinct experimental set-ups [10]; they are thus indicative of presence of these proteins in the two datasets but are not meant as quantitative information on protein abundance in these cells types. Typical components of an axoneme are clearly identified. **(B)** Longitudinal section of an axoneme and basal body and the proteins identified in the male proteomes that are putatively associated with these structures. **(C)** Proteins that were identified in the male gamete proteomic analysis but that could not be attributed to a specific compartment of the flagellum, these proteins possess bio-informatic prediction that suggests they could play a role in flagellar biology. CPP = conserved *Plasmodium* protein.

The flagellum is amongst the most conserved of eukaryotic cytoskeletal structures. Electron microscopic observations on flagella in *Plasmodium* and other Haemosporida [1, 7, 72–74] suggest:

-  Axoneme structure conforms to the typical 9 + 2 pattern;
-  The basal bodies/centrioles lack a B and C-tubule;
-  An outer arm dynein can be observed on most microtubule doublets;
-  Inner dynein arms are less frequently observed and they appear to be less electron dense than the outer arm;
-  No nexin links between microtubule doublets have been described;
-  The central apparatus is conserved at the morphological level, containing the central microtubule pair and radial spokes.Several major families of proteins identified in the eukaryotic flagellum are widely conserved (e g, tubulins, dyneins and kinesins). Within the microgamete proteome members of these protein families and the accessory proteins are presented in the context of their putative function and expression profile (Figure 3).

All major tubulins are present in the *Plasmodium* genome, including two subtypes of α tubulin. Α and β types form dimers that polymerize in linear arrays in microtubules, which in turn are organized/patterned by basal bodies to form axonemes. Both forms of α-tubulin and β-tubulin were found in the male gamete proteome suggesting that both α-tubulin subtypes participate in axonemal formation. The microtubule organizing centre (MTOC) tubulin types (e g, Δ,δ and ϵ) were not identified in the male gamete proteome. This is surprising since δ and ϵ type functions in other systems are intrinsically linked to centriole/basal body biology and male gametes are reportedly the only stage possessing a centriole [75].

Dynein arm complexes are composed of a variable number of heavy, intermediate and light chains. In this study, three outer arm heavy chains (two OADβ and one OADα) as well as two outer arm intermediate chains (IC70 and IC78) were found. This strongly suggests that, as has been hypothesized by Wickstead and Gull [4], the *Plasmodium* outer dynein arm is three-headed.

IAD-β was found in the male gamete and inner dynein arms are seen in electron micrographs of *Plasmodium* axonemes. Nevertheless the absence of annex components of a *bona fide* IAD (e g, intermediate chains 138 and 140) in the genome suggests a divergent organization of the inner arm complex.

Surprisingly only three dynein light chains were found in the male gamete proteome: LC1, LC8 and LC14. LC8 has been associated with the OAD complex [76] and found to interact with radial spokes [77]; LC1 was shown to be important for outer dynein arm function in eukaryotic flagella [78] but was also show to have divergent function in *Plasmodium* erythrocytic stages [79]; the function of LC14 is not known. Light chains are important in the recruitment and assembly of motor components and motor function itself [80, 81]. The possible under-representation of light chains in the male gamete proteome raises the appealing hypothesis that the light chains play a role in axoneme assembly in the gametocyte cytoplasm but are dispensable for flagellum function in the male gamete.

Three kinesins are found in the male gametocyte and the male gamete. It is difficult to speculate on the function of these proteins as kinesins also play a key role in mitosis. The microgamete kinesins identified include Kinesin-8, Kinesin-13 and an ungrouped kinesin [82]. Kinesin-8 family members have been implicated in microtubule depolymerization [83, 84], notably in the mitotic spindle and could therefore be contained in the nucleus. Kinesin-13 has been implicated in flagellar length control in a variety of organisms [85]. Moreover kinesin molecules have been associated with the central doublet pair and may play a role in flagellar regulation [86, 87].

The central microtubule doublet apparatus has been implicated in the regulation of flagellar beating [88]. The accessory proteins PF16, PF20, actin and actin related-proteins, calmodulin and some conserved radial spoke proteins (Figure 3) were found in the male proteomes. PF16 was been shown to a key component for the assembly of the central apparatus of the *P. berghei* axoneme [61].

Despite having been reliably identified in the *Plasmodium* genome [4] none of the components of the cytoplasmic dynein complex (e g, cytoplasmic dynein heavy chain PBANKA_021400, cytoplasmic light chains PBANKA_041490 and PBANKA_082830), which normally engage in cargo transport along both cytoplasmic and flagellar microtubules, was found in the proteome (see Additional file 3), suggesting there is no transport of dynein-dependent cargo along the length of the male gamete axoneme.

Taken together, these observations suggest that the *Plasmodium* axoneme, in spite of possessing numerous highly conserved structures, is characterized by relatively few conserved core proteins. Two biological features may account for this observation: first, axoneme assembly occurs in the cytoplasm, the regulators of assembly may therefore be divergent and may not be carried over into the flagellum; second, the remarkably short life span of a male gamete might suggest any ‘maintenance apparatus’ of the flagellum is minimal or indeed absent.

## Conclusions

This is the first analysis of the proteomic content of an apicomplexan male gamete and opens new avenues of research both into the unusual organization and biology of the *Plasmodium* gamete and may underpin new possibilities by which to target male gametes as a transmission blocking strategy.

## Electronic supplementary material

Additional file 1: **Protein identified in the male gamete proteome.** Description: List and attributes of the 615 proteins found in this study. (PDF 107 KB)

Additional file 2: **Gene ontology analysis.** Description: GO terms enrichment test in the proteome. (PDF 299 KB)

Additional file 3: **Generation and validation of PbHT-myc strain.** Description: Recombination strategy and validating Southern blot of transgenic parasites. (XLSX 126 KB)

Additional file 4: **Validation of spectra and peptide identification.** Description: Receiver Operating Characteristic (ROC) of total spectra and total peptide identifications from multiple search pipeline.) (XLSX 39 KB)

Additional file 5: **A male gamete displays both fast and slow types of flagellar beat Description: Scale bar 5 μm.** (AVI 1 MB)
