# Supplementary material for: Proteomic analysis of the Plasmodium male gamete reveals the key role for glycolysis in flagellar motility
Source: Malar J. 2014 Aug 13;13:315. doi: 10.1186/1475-2875-13-315 (PMC4150949; doi:10.1186/1475-2875-13-315)
Supplement: Supplementary file 1 — Additional file 1: Protein identified in the male gamete proteome. Description: List and attributes of the 615 proteins found in this study. (PDF 107 KB) [file 12936_2014_3362_MOESM1_ESM.pdf]

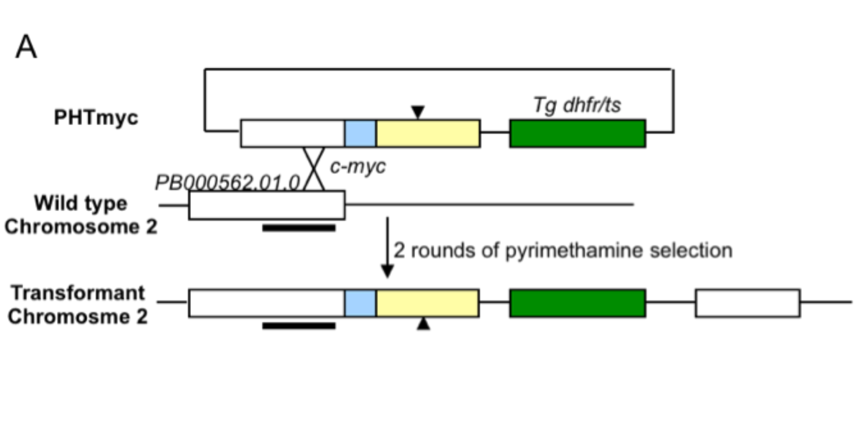

(A) Integration strategy of *pHTmyc* in chromosome 2 of *P. berghei.* Following single cross-over, the endogenous hexose transporter locus (white) is modified to contain a C-terminal c-myc-tag (blue) followed by the *P. berghei dhfr* 3'UTR (yellow). Integration is selected by pyrimethamine resistance conferred by the *Toxoplasma gondii dhfr/ts* gene (green). The heavy black bars indicate the location of the probe for southern blotting. Arrowheads represent the HindIII restriction sites used for genomic DNA digestion for southern blotting. (B) Southern blot of genomic DNA from wild type (WT) and transformant (HT) parasite populations. Upon integration, a HindIII restriction site from the plasmid is inserted into the HT locus resulting in a signal at 3kb (black arrowhead) as opposed to the wild type signal at >10kb (white arrowhead).
