# Supplementary material for: Proteomic analysis of the Plasmodium male gamete reveals the key role for glycolysis in flagellar motility
Source: Malar J. 2014 Aug 13;13:315. doi: 10.1186/1475-2875-13-315 (PMC4150949; doi:10.1186/1475-2875-13-315)
Supplement: Supplementary file 2 — Additional file 2: Gene ontology analysis. Description: GO terms enrichment test in the proteome. (PDF 299 KB) [file 12936_2014_3362_MOESM2_ESM.pdf]

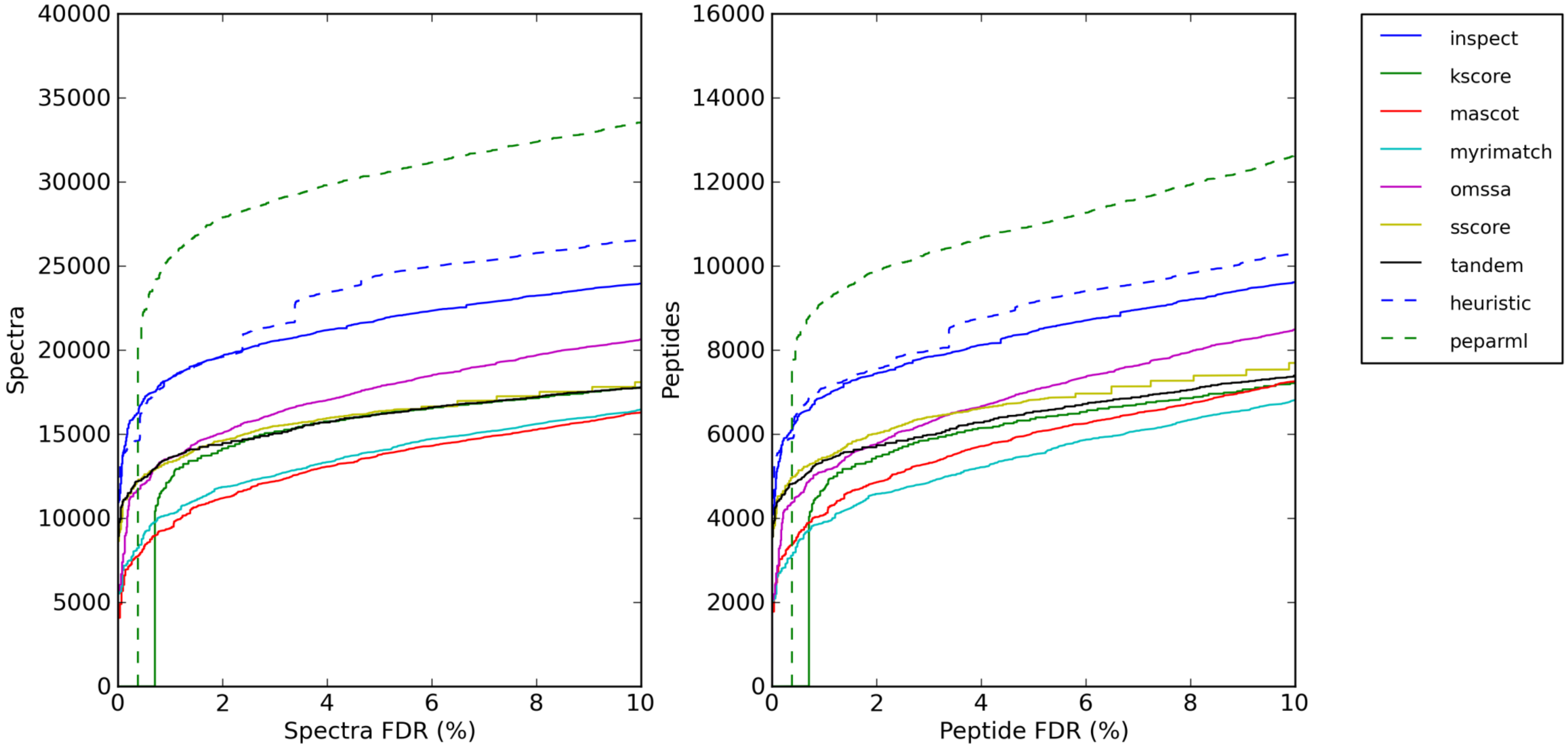


ROC of total spectra and total peptide identifications from multiple search pipeline: Spectra and Peptide FDR calculations from multiple search engines are represented with different colors. Heuristic and PepArML combiner performance from the meta search results. PepArML combiner results were selected for 1% spectra and 1% peptide FDR. Proteins from human and mouse, and proteins with shared peptides, single peptide hits, and peptides with less than 5 amino acids were later excluded from analysis by Maspectras.
